# A database to analyze cycling routes in Medellin, Colombia

**DOI:** 10.1016/j.dib.2020.106162

**Published:** 2020-08-08

**Authors:** Juan P. Ospina-Zapata, Víctor I. López-Ríos, Verónica Botero-Fernández, Juan C. Duque

**Affiliations:** a Universidad Nacional de Colombia, Sede Medellín, Colombia; b Research in Spatial Economics (RiSE-Group), Department of Mathematical Sciences, Universidad EAFIT, Colombia

**Keywords:** Cycling, Survey, Travel behavior, Cycling routes, Database

## Abstract

A bicycle route questionnaire was designed to collect information about the characteristics of cyclists and the routes they take. Medellin is used as a case study in this paper due to its strong sociodemographic inequality, land use, urban form diversity, and topographical variability. The survey execution targeted bicycle commuters in the city by distributing the questionnaires online, personally by telephone, and personally on the street. These data will be useful to support strategies aiming to promote bicycling as a mode of transportation. Several types of analysis may be derived from the data, including an explanation of the factors determining the route choice and route comparisons according to the sociodemographics and locations of users. For instance, these data have already been used by Ospina et al. (2020) [1] where they sought to understand cycling travel distance in Medellin city.

**Specifications Table**

**Table:** 

**Subject**	Transportation
**Specific subject area**	Cycling travel behavior.
**Type of data**	Table Shape file Document text Jupyter Notebook
**How data were acquired**	A bicycle route questionnaire was designed to collect data containing information about the characteristics of cyclists and detailed information related to the routes they take. The survey execution targeted current bicycle commuters in Medellin, Colombia by distributing the questionnaires online, personally by telephone and personally on the street. The online version of the questionnaires was distributed randomly. However, because the online version only covered a certain type of population, we used the surveys that were delivered personally by telephone and personally on the street to target some “lacking” profiles, which provided a representation of different cycling profiles. Sections 3 and 4 detail more about the sampling construction and the data collection.
**Data format**	**Raw data** Document text: word file Table: excel file **Secondary data** Shape file: ESRI shape file **Processed data** Shape file: ESRI shape file Python codes
**Parameters for data collection**	The Medellin Metropolitan Area authority (Área Metropolitana del Valle de Aburrá - AMVA) conducts the Origin-Destination survey (ODS), which estimates that the proportion of regular cyclists in the population is relatively small (1%) [2]. The ODS uses a traditional methodology based on random sampling from all the “traveling” population in the city. However, when a particular focus on cyclists is required, this methodology has several limitations: 1) it has a limited representation of the cycling population, 2) the spatial distribution of cyclist sampling is limited, 3) it does not include route path information, and 4) it has limited attributes associated with cyclists [3]. Therefore, we applied a mixed sample selection by using the online survey in a random way, and the telephone and street surveys in a nonrandom way. Sections 3 and 4 detail more about the sampling construction and the data collection.
**Description of data collection**	The survey was conducted from February 2017 to March 2017, which is considered a regular-season for studying and working. The questionnaires were distributed online, personally by telephone and personally on the street. From all the respondents, we selected current cyclists who could accurately recall their routes, which was the main goal of our survey. Consequently, we surveyed 810 cyclists whose routes were geocoded and then matched to a street network. Finally, the physical variables corresponding to the routes were generated using scripts written in the Python programming language for Qgis. Sections 4 details the data collection.
**Data source location**	Institution: Universidad Nacional de Colombia, Sede Medellin City/Town/Region: Medellin Country: Colombia Latitude and longitude (and GPS coordinates) for collected samples/data: See section 6 for details of the sampling distribution. The related sample coordinates are included in the shape file associated with the trip origin points. **Primary data:** Questionnaire. *File Name:* Ospina_cyclists_questionnaire.docx Questionnaire responses of each of the 810 cyclists in Medellin. ***File Name:*** Ospina_QuestionResponses_DIB.xlsx. **Processed data based upon the primary and secondary information:** Database including all the variables associated with the routes of each of the 810 cyclists in Medellin*.****File Name:*** Ospina_cyclists_BD_DIB.xlsx
**Data accessibility**	**Mendeley Data repository:** https://data.mendeley.com/datasets/w22y5zk9kh/1 Ospina, Juan Pablo; López-Ríos, Víctor I.; Botero-Fernández, Verónica; Duque, Juan C. (2020), “Database Cyclists. Medellin, Colombia ”, Mendeley Data, V2, DOI: 10.17632/w22y5zk9kh.1 (The database is already public) This database includes: **Primary data:** Questionnaire. ***File Name:*** Ospina_cyclists_questionnaire.docx Questionnaire responses of each of the 810 cyclists in Medellin. ***File Name:*** Ospina_QuestionResponses_DIB.xlsx. **Processed data based upon the primary and secondary information:** Database including all the variables associated with the routes of each of the 810 cyclists in Medellin*.****File Name:*** Ospina_cyclists_BD_DIB.xlsx All shapes that include processed data based upon the primary and secondary information (See data section for more details) **Secondary data:** Shape file including the Medellin administrative zones and comunas. Six administrative macrozones into which Medellin City is divided. These aggregate the comunas. Medellin counts 16 comunas. **File Name:** Ospina_MDE_zones_wgs84.shp Shape file including the municipalities belonging to the metropolitan area. **File Name:** Ospina_AMVA_municipios_wgs84.shp Shape file including the urban perimeter of Medellín city. **Filename:** Ospina_MDE_perimeter_wgs84.shp
**Related research article**	Authors’ names: Juan P. Ospina-Zapata, Verónica Botero-Fernández, Juan C. Duque, Mark Brussel, A. Grigolon Title: Understanding cycling travel distance. The case of Medellin city (Colombia) Journal: Transportation Research Part D. DOI: 10.1016/j.trd.2020.102423

**Value of the Data**

Cities are seeking to raise cycling levels and improve the safety of bicycle users, for which understanding the travel behavior of cyclists is essential. Therefore, the data contained in the current database will be useful to determine 1) who cycles by characterizing current bicycle users, and 2) where they cycle by tracing the routes they take.
These data will benefit urban planners and stakeholders working on the design and implementation of cycling infrastructure in response to the current behavior of cyclists. Also, it will be useful for researchers aiming to design strategies to motivate new bicycle users, improve cycling safety, and make cycling possible for different segments of society.
In developing countries where resources are limited, different strategies are undertaken to capture information, from cyclists in this case, while considering cost limitations. A non-expensive tool for surveying bicycling routes is useful to support strategies concerning bicycle promotion in developing countries.
Different elements make Medellin an interesting case to be studied with the aim of better understanding the travel behavior of cyclists. The city is characterized by the diversity of urban environments, high levels of socioeconomic inequality, and high topographical variability.
Several types of analyses can be derived from the data including an explanation of the factors that determine the route choice, route comparisons according to sociodemographic features and location, design of bicycle networks, accessibility analysis of the main urban activities for cyclists, innovative methodologies to capture data from cyclists and others.

## 1 Data

1. **Tables included in the Annex (Folder: Tables)**:

**Table:** 

Type of data	File name	Description
Raw data	Ospina_cyclists_BD_DIB.xlsx	Database including all the variables associated with the routes of each of the 810 cyclists in Medellin.
Raw data	Ospina_QuestionResponses_DIB.xlsx	Questionnaire responses of each of the 810 cyclists in Medellin.
Raw data and secondary information	Table only included in this paper (no file). Name of the table: Table 1*. Distribution of the sampling framework according to zone and income.*	Sample and Origin-Destination Survey (2012) distributions according to Medellin zone and income strata. Based upon [1] and complemented with secondary information from [2].

2. **Figures included in the text and the Annex (the file names are the same as in the list) (Folder: Figures)**:

**Table:** 

Type of data	File name	Description
Secondary data	Fig. 1 included in this paper. Spatial distribution of 6 macrozones in Medellin. File name:	Spatial distribution of 6 administrative macrozones in Medellin, which are defined according to the location and the average socioeconomic characteristics of the population.
Raw data and secondary information	Fig. 2 included in this paper. Distribution of our sample framework according to the macrozones in relation to the ODS sampling.	Distribution of our sample framework according to the macrozones in relation to the ODS sampling.
Raw data and secondary information	Fig. 3 included in this paper. Distribution of our sample framework according to income in relation to the ODS sampling.	Distribution of our sample framework according to income in relation to the ODS sampling. The secondary information is based on [2].

3. **Shape files included in the database repository (Folder: Shapes/Subfolders associated with the name in this list)**:

**Table:** 

Type of data	File name	Description
Processed data based upon secondary information	Red_PhD_arcs_20,181,027.shp	Shape file including the OSMN Medellin street network (arcs).
Processed data based upon secondary information.	Red_PhD_nodes_mallavial.shp	Shape file including the OSMN Medellin street network (nodes).
Processed data based upon primary and secondary information.	Ospina_cyclists_routes_wgs84.shp	Shape file including the 810 paths (dissolve). The ID of each route allows linking the data from the database.
Processed data based upon primary and secondary information.	R_ID_phd_arcs_VF.shp	Shape file per each of the 810 paths (arcs). The ID of each route allows linking the data from the database. (the ID corresponds to the route ID code. For instance, for the first route in the list, the shape file would be R1002_phd_arcs_VF.shp.
Processed data based upon primary and secondary information.	R_ID_phd_route_nodes.shp	Shape file including the 810 paths (nodes). The ID of each route allows linking the data from the database.
Processed data based upon primary data.	Ospina_cyclists_origins_wgs84.shp	Shape file including the 810 trip origins. The ID of each route allows linking the data from the database.
Processed data based upon primary information.	Ospina_cyclists_destinations_wgs84.shp	Shape file including the 810 trip destinations. The ID of each route allows linking the data from the database.
Secondary data.	Ospina_MDE_zones_wgs84.shp	Shape file including the Medellin administrative zones and comunas. Six administrative macrozones into which Medellin City is divided. These aggregate the comunas. Medellin counts 16 comunas.
Secondary data.	Ospina_AMVA_municipios_wgs84.shp	Shape file including the municipalities belonging to the metropolitan area.
Processed data based upon primary and secondary information.	R_ID_start_arcs600.shp	Shape file including the street segments within a 600-m buffer around the trip origin. The ID corresponds to the route ID code.
Processed data based upon primary and secondary information.	R_ID__end_arcs600.shp	Shape file including the street segments within a 600-m buffer around the trip destination. The ID corresponds to the route ID code.
Processed data based upon primary and secondary information.	R_ID_ start_encicla600.shp	Shape file including the bike-sharing system (Encicla) station points within a 600-m buffer around the trip origin. The ID corresponds to the route ID code.
Processed data based upon primary and secondary information.	R_ID_end_encicla600.shp	Shape file including the bike-sharing system (Encicla) station points within a 600-m buffer around the trip destination. The ID corresponds to the route ID code.
Processed data based upon primary and secondary information.	R_ID_ start_intersections600.shp	Shape file including the street intersection points within a 600-m buffer around the trip origin.
Processed data based upon primary and secondary information.	R_ID_end_intersections600.shp	Shape file including the street intersection points within a 600-m buffer around the trip destination. The ID corresponds to the route ID code.
Processed data based upon primary and secondary information.	R_ID_ start_LU600.shp	Shape file including the land use polygons within a 600-m buffer around the trip origin. The ID corresponds to the route ID code.
Processed data based upon primary and secondary information.	R_ID_ end_LU600.shp	Shape file including the land use polygons within a 600-m buffer around the trip destination. The ID corresponds to the route ID code.
Processed data based upon primary and secondary information.	R_ID_route_LU100.shp	Shape files including the land use polygons along the 100-m buffer along the route. The ID corresponds to the route ID code.
Secondary data.	Ospina_MDE_perimeter_wgs84.shp	Shape file including the urban perimeter of Medellín city.

4. **Document text included in the database repository**: **(Folder: Codes**:

**Table:** 

Type of data	File name	Description
Raw data	Ospina_cyclists_questionnaire.docx	The final version of the questionnaire used to survey cyclists by telephone, online, and on the street.
Raw data	Ospina_route_list.txt	List of the 810 ID routes.

5. **Jupyter notebook** (Python) codes:

**Table:** 

Type of data	File name	Description
Processed data based upon secondary information.	Code_Networkx_OSMNx_MEDELLIN OPEN STREET DATA.ipynb	Jupyter Notebook (Python) file code to download Medellin's Street Network from OSMNx.
Processed data based upon primary and secondary information.	105_O_start_intersections_INDEX analysis.ipynb 106_D_end_intersections_INDEX analysis.ipynb	Jupyter Notebook (Python) file code for the Origin and Destination variables related to the intersections.
Processed data based upon primary and secondary information.	107_O_start_street density_analysis.ipynb 108_D_end_street density_analysis.ipynb	Jupyter Notebook file code for the Origin and Destination variables related to the street density.
Processed data based upon primary and secondary information.	109_O_start_arcs infrastructure_analysis.ipynb 110_D_end_arcs_infrastructure analysis.ipynb	Jupyter Notebook file code for the Origin and Destination variables related to the infrastructure of the street segments.
Processed data based upon primary and secondary information.	113_O_start _LU polygons_analysis.ipynb; 114_D_end _LU polygons_analysis.ipynb	Jupyter Notebook file code for the Origin and Destination variables related to the Land Use.
Processed data based upon primary and secondary information.	123_OD_encicla_bsindex.ipynb	Jupyter Notebook file code for the Origin and Destination variables related to the stations of the bike-sharing system.
Processed data based upon primary and secondary information.	202_Route Intersection INDEX Analysis.ipynb	Jupyter Notebook file code for the route variables related to the intersections.
Processed data based upon primary and secondary information.	212_Route_LU polygons analysis.ipynb	Jupyter Notebook file code for the route variables related to land use.
Processed data based upon primary and secondary information.	221_Route_Infrastructure analysis.ipynb	Jupyter Notebook file code for the route variables related to the infrastructure of the segments.

## 2 Experimental design, materials, and methods

Current urban agendas are seeking to promote bicycle use to improve the sustainability of cities and transportation systems. Thus, national and local governments are implementing complementary measures to raise cycling levels and improve the safety of bicycle users [4]. A key aspect with direct influence over bicycle use is the existence of dedicated infrastructure along cycling paths. In this context, planners must design routes in such a way that cycling is possible for cyclists who are highly trained as well as for those who are just starting [4]. The provision of data that facilitates an understanding of the travel behavior of cyclists is essential for raising cycling levels and improving the safety of bicycle users [4].

Conventional transport demand surveys are commonly used to analyze general mobility patterns including all modes of transport. Consequently, they might not give accurate information when details are required for a specific segment of the population or a transportation mode such as cycling [5]. The Medellin Metropolitan Area authority (Área Metropolitana del Valle de Aburrá - AMVA) conducts the Origin-Destination survey (ODS), which estimates that the proportion of regular cyclists in the population is relatively small (1%) [2]. The ODS uses a traditional methodology based on random sampling from all the “traveling” population in the city. However, when a particular focus on cyclists is required, this methodology has several limitations: 1) it has a limited representation of the cycling population, 2) the spatial distribution of cyclist sampling is limited, 3) it does not include route path information, and 4) it has limited attributes associated with cyclists [3].

The database presented in this paper relies on information derived from a survey targeting current bicycle commuters who were recruited by using a mixed random and nonrandom method to obtain a diversity of cyclist profiles. This section of the paper first explains what makes Medellin an interesting case of study. The second section presents the list of cyclist attributes and the physical characteristics of the routes to be surveyed. In addition, the third part details the questionnaire preparation. The fourth part describes the construction of the sampling framework, and the fifth part explains how the cyclists’ data were collected. The sixth part details the characteristics of the street network used and the route geocoding process. Finally, the seventh part presents our sampling distribution, which is compared with the previous metropolitan survey sampling.

### 2.1 Why Medellin?

Medellin is recognized worldwide because of the recently implemented measures favoring public transportation and urban development. However, despite the implementation of several strategies to increase bicycle use as a mode of transport, cycling remains a big challenge in the city. Therefore, different elements make Medellin an interesting case to be studied with the aim of better understanding the travel behavior of cyclists. First, local governments have implemented measures to raise cycling levels in Medellin, especially during the last decade. Despite improvements to the dedicated infrastructure and the bike-sharing system, commuting cycling levels remain very low (1%) [2]. Second, considering its geographical location in the Northern Andes of Colombia, Medellin is surrounded by mountains with altitudes ranging from 1500 m to 2500 m above sea level. Consequently, urban slopes range from 0% to more than 20%, which implies extra efforts for urban cyclists. Third, the central part of the city is well supplied in terms of services, in contrast with the urbanization towards the periphery, both legally and illegally, where a lack of infrastructure, services, and public facilities is evident. As a result, urban activities and jobs are located very far from where the populations are located in the northern and western parts of the city. Fourth, legal and illegal urbanization have shaped the city and its street networks, which may determine the type of routes that cyclists take. While the former gave place to regular street shapes, the latter produced organic structures of street networks following the shape of the topography. Fifth, the concentration of a low-income population in the northern part of the city and a high-income population in the southern part shows that Medellin has a high level of social inequity [6,7].

### 2.2 Definition of variables

Cycling behavior for commuting purposes is determined by several factors including the attributes of individuals and the physical characteristics of the routes they take [8,9]. To define the set of characteristics to be analyzed in our study, we first identified a set of attributes that we considered to be especially relevant according to the literature [8], [9], [10], [11]. We classified these attributes into three dimensions: 1) individual, 2) built environment related to the origin and destination, and 3) built and natural environment related to the route [8,9]. Once we had defined the characteristics to be studied, we prepared a set of questions to obtain information about individuals and their routes. Please refer to Ospina et al. (2020) [1] for a full description of the variables included in each of the dimensions. These definitions correspond to the variables contained in the database presented in this paper.

### 2.3 Questionnaire preparation

Based on the variables, we designed a questionnaire to obtain the characteristics of current bicycle users. The questionnaire went through several drafts and pilots before its final distribution. The first draft of questions was discussed with experts from the metropolitan bike-sharing system (Encicla) and local cycling advocacy organizations. Subsequently, the first pretest pilot of the questionnaire was distributed to 20 current cyclists who filled it out and gave us their comments. The questionnaire was modified according to the suggestions from both revisions. As a result, we selected those questions that would elicit the most essential information from cyclists during interviews lasting at most 10 min.

The final version of the questionnaire (see Annex) contained 25 five questions organized into three sections. The first section was intended to identify current bicycle commuters from commuters using other transportation modes. This section was designed especially for online respondents considering the difficulty of determining in advance who would receive the questionnaire. The second section, which was only applicable to current cyclists, focused on basic sociodemographics and some general travel information. The third section complemented the previous questions with a description of the routes taken by cyclists to do their last trip when commuting to work or to study.

### 2.4 Sampling framework construction

**Fig. 1:**
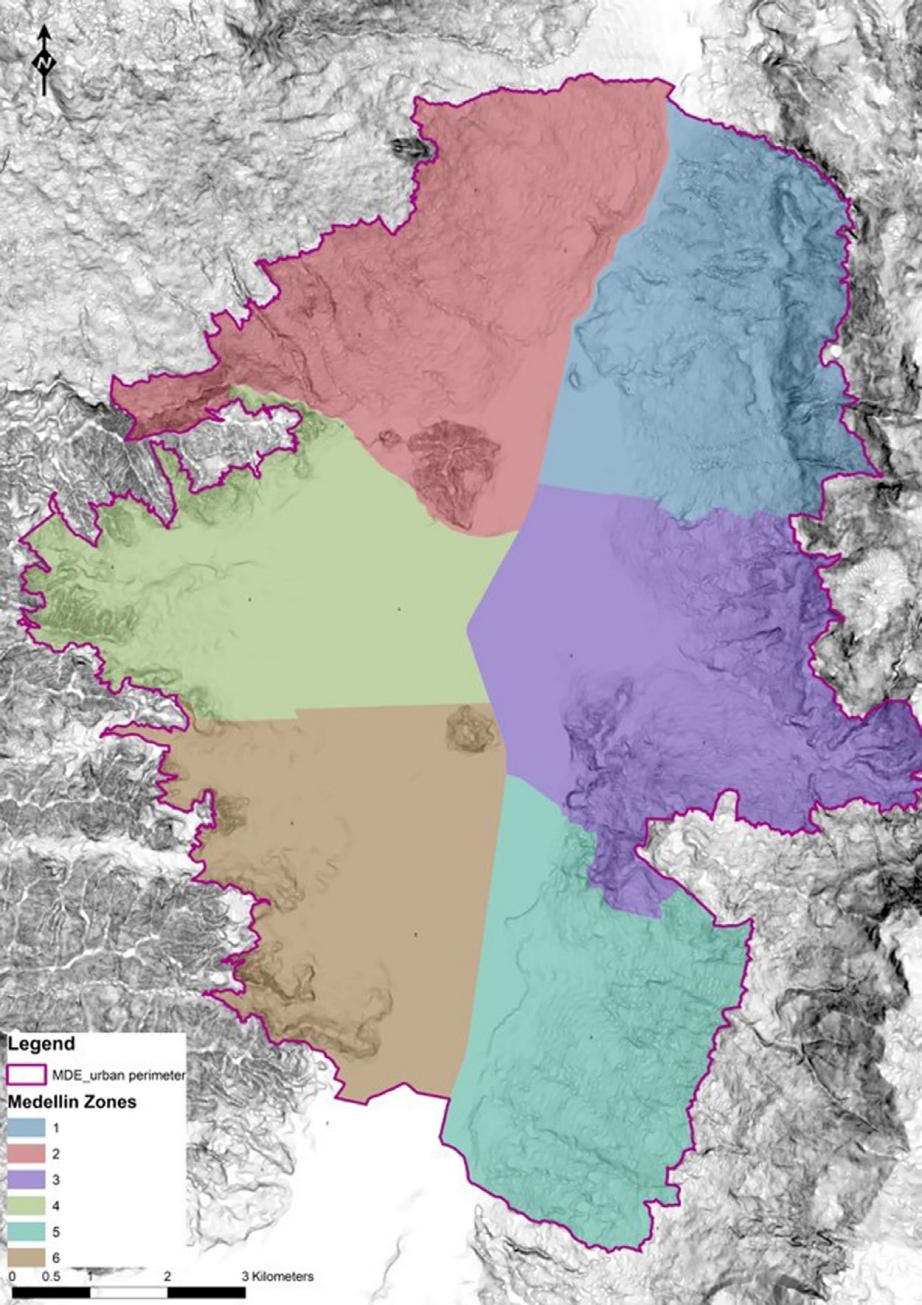
Spatial distribution of 6 macrozones in Medellin.

To identify a set of bicycle users for our study, we first tried to contact the cyclists reported in the Origin-Destination survey (ODS) database [2]. However, we were only able to contact 4% of them, which was not enough to build a robust sample of cyclists. Therefore, we explored other alternatives. First, a simple random method was initially discarded, given that it would be very inefficient to find enough cyclists due to the small proportion of the cycling population [3]. We then explored a random stratified sampling method composed of two stages: 1) the population is partitioned into nonoverlapping groups called strata, and 2) a random sample is selected by some design within each stratum [12]. For the first stage, we divided the territory into six strata (groups)^1^ according to the six administrative macrozones into which Medellin is divided. Every macrozone is an aggregation of several neighborhoods that usually share similar income levels.^2^ Fig. 1 shows the spatial distribution of the six macrozones, which we kept from the first stage of the stratified sampling method. The first and second zones, located in the northern part of the city, are characterized by a low-income-level population and a hilly topography. The third zone is located in the eastern central part of the city and includes the downtown; its topography is moderate and is mainly occupied by a medium-income-level population. The fourth zone is located in the western central part of the city; its slopes are mostly under 6% with some hills towards the peripheries, and it contains a diversity of income levels. The fifth zone is located in the southeastern part of the city, which is mainly occupied by a high-income-level population, and the topography is mostly hilly. Finally, the sixth zone, located in the southwestern part of the city, is characterized by a medium-income-level population, and the topography is mostly flat.

**Fig. 2:**
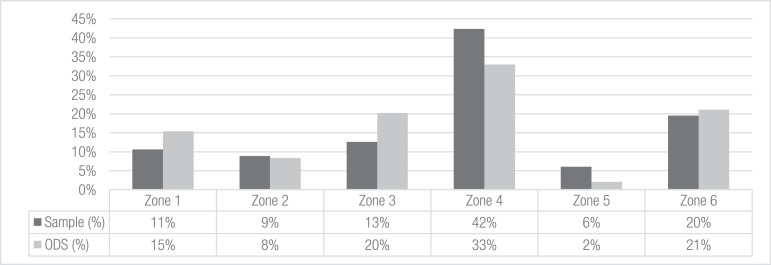
Distribution of our sample framework according to the stratified sample zones (dark columns) in relation to the ODS sampling (gray columns). Based upon [1] and complemented with secondary information from [2].

Concerning the second stage of the stratified method, despite estimating the total population from the total cycling trips generated from each stratum according to the ODS, the random selection of cyclists was inefficient or even inaccurate considering two factors. First, it was inefficient because of the small share of cyclists within each stratum, which would make it very hard to find enough cyclists. Second, it was inaccurate because the ODS concerns the total population of daily travelers, including all modes and all types of purposes, and we were only focused on a specific group of cyclists.

Considering these limitations, the stratified method was partially used. To have a starting point for efficiently identifying the targeted cycling population, we kept the six strata derived from the first phase. We later identified the sociodemographic profiles of cyclists in each stratum based on the ODS database. We chose the income level as a key feature to define the cyclist profiles to be targeted from each stratum. We then applied a mixed random and nonrandom selection from each stratum. That is, the online version of the questionnaire was distributed randomly, and therefore, the sample extracted from these responses was randomly selected. However, because the online version only covered a certain type of population, we distributed surveys personally by telephone and on the street to target some “lacking” profiles, which provided a representation of different cycling profiles. Consequently, the sample extracted from the personally distributed questionnaires was conducted in a nonrandom way. Section 4 details the data collection process.

### 2.5 Data collection

The survey was conducted from February 2017 to March 2017, which is considered a regular-season for studying and working. The questionnaires were distributed online, personally by telephone and personally on the street. The online version was distributed by e-mail through universities, public administration offices from the Medellin metropolitan area, and cycling advocacy groups. With respect to the phone surveys, some of the cyclists were contacted based on the prior ODS database, and others were reached through the actors involved in the online version of the survey. The process was different for the street surveys. Based on previous metropolitan bicycle studies [13], we first identified the best locations to perform the surveys according to three conditions: 1) the corridors associated with a high concentration of bicycle commuters, 2) the intersections over the main corridors coinciding with traffic lights where cyclists usually stop, and 3) some other locations with a lower concentration of cyclists but coinciding with traffic lights, which were also highlighted in previous bicycle studies. The interviewers were strategically distributed at locations where they could take advantage of cyclists stopping and easily get their attention to conduct the survey.

Once started, a revision was completed every week during the period of the survey. Such revisions consisted of identifying the respondents’ starting trip location and income level. We then compared such information with the profiles obtained for each stratum (macrozone) in the ODS sampling. This verification advised us about the over/underrepresentation of certain cyclist profiles, which let us know in advance the type of profiles to focus on every week. Because we did not have much control over the distribution of the online version, we used the telephone and street surveys to target such profiles. Even though we did not pretend to achieve the same ODS sampling distribution for each stratum, this verification helped to guarantee the diversity of cyclists and avoid the possible concentration of cyclists belonging to certain strata. This explains why our methodology was a mixture of random and nonrandom sampling. Section 6 details the final distribution of our sampling and compares it to the one obtained by the ODS.

The survey involved 2760 respondents, with 93% from online surveys, 5% from telephone surveys, and the remaining 3% from personal interviews on the street. A total of 1835 (66%) out of 2760 people were current cyclists, and the remaining 925 were users of other transportation modes. Most of the non-bicycle users came from online surveys (889) due to the difficulty of filtering them out before the distribution of the questionnaires. The remaining non-bicycle users (36) were contacted by telephone. This justifies designing the first section of the questionnaire to select current bicycle users from commuters using other modes of transportation.

**Fig. 3:**
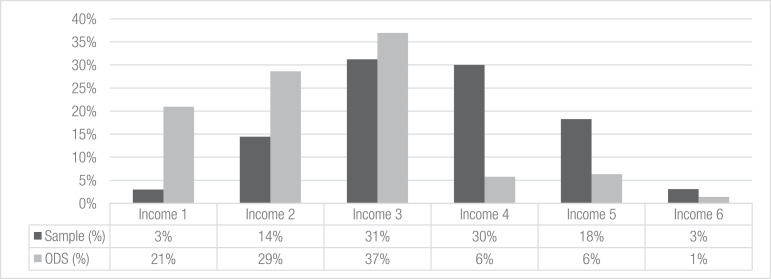
Distribution of our sample framework according to income in relation to the ODS sampling. Based upon [1] and complemented with secondary information from [2].

We finally selected those current cyclists who could accurately recall their routes, which was the main goal of our survey. Consequently, we kept 810 of the 1835 cyclists whose route descriptions enabled precise geocoding (see Fig. 3). Missing street names and nonlogical descriptions were common mistakes among the excluded routes, which mostly arrived from online surveys. Such mistakes were less recurrent in the phone and street surveys, given that the interviewers were always present to ask for detailed information to achieve accurate route descriptions. The selected 810 respondents are the ones contained in the current database, which includes all the variables that have already been described.

### 2.6 Street network and routing geocoding

The 810 routes were geocoded and then matched to a street network, which was compiled from three different sources. The first source was the Open Street Maps Network (OSMN), which included the basic characteristics of the roads. This network, composed of a set of very well-connected nodes and links, was downloaded using OSMNx [14] and the Python programming language script, which is included in the Annex. The second source was the metropolitan street network, which included updated information about the hierarchy of the roads. The third source was the metropolitan bicycle network, which had all current (the year 2017) segments including the cycling infrastructure. The merger of all three sources resulted in a detailed network, which is available in the Annex, covering the entire urban context of Medellin and containing the physical characteristics of the road segments. The selected 810 routes were first traced on Google Maps and then matched to the street network links by using QGIS. Finally, the physical variables corresponding to the routes were generated using scripts written in the Python programming language for Qgis. These scripts and the shape file containing the 810 routes are also included in the Annex.

### 2.7 Sampling distribution

This section shows the spatial distribution of the 810 cyclists and compares the sampling distribution to the one obtained by the ODS. The resulting sampling (*N* = 810 cyclists) represented 44% of the current bicycle users who responded to the survey and were distributed throughout the city of Medellin. The spatial distribution of the 810 trip origins as well as the routes are detailed in [1].

With respect to the sampling distribution, Fig. 2 shows that the cyclists came from all six strata groups defined in the first stage of the stratified method. As explained in the previous section, some differences were previously expected to result from a comparison to the ODS because we did not pretend to achieve the same ODS sampling distribution for each stratum. As shown in Fig. 2, some of the zones of our sample had more representation, while others were less represented compared to the ODS. Similarly, there were also some differences between the ODS and our sample regarding the income level distribution shown in Fig. 3. In addition to the sample limitations already described in section 4, several elements explain these differences. First, a small number of cyclists belonging to the 1st income level was captured by the online surveys in the institutions where the questionnaires were distributed. Despite using the phone and street surveys to obtain seemingly underrepresented profiles, the budget and time restrictions limited the possibility of surveying more cyclists. Second, regarding the 5th income level, the fact that bicycles are not frequently used as a mode of transport may explain its low representation. Finally, Table 1 summarizes the data shown in Figs. 2 and 3 according to zone and income.

**Table 1:** Sample and ODS distributions according to Medellin zone and income strata. Based upon [1] and complemented with secondary information from [2].

	Income 1	Income 2	Income 3	Income 4	Income 5	Income 6	TOTAL	Sample (%)	ODS (%)
Zone 1	12	46	26	2			86	11%	15%
Zone 2	2	18	41	8	3		72	9%	8%
Zone 3	2	16	50	29	3	2	102	13%	20%
Zone 4	6	26	68	149	89	5	343	42%	33%
Zone 5		2	2	5	23	17	49	6%	2%
Zone 6	2	9	66	50	30	1	158	20%	21%
TOTAL	24	117	253	243	148	25	810		
Sample (%)	3%	14%	31%	30%	18%	3%			
ODS (%)	21%	29%	37%	6%	6%	1%			

## 3 Special advice

Considering that our sample was obtained through a mixture of random and nonrandom sampling methods, the conclusions derived from this database must refer to our sampling method, and although such conclusions may be coincident, no generalizations can be drawn for the entire population of Medellin.

## Ethics statement

Hereby, I Juan Pablo Ospina Zapata, and also on behalf of my co-authors, consciously assure that for the manuscript “A database to analyze cycling routes in Medellin, Colombia” the following is fulfilled:

1) This material is the authors' own original work, which has not been previously published elsewhere.
2) The paper is not currently being considered for publication elsewhere.
3) The paper reflects the authors' own research and analysis in a truthful and complete manner.
4) The paper properly credits the meaningful contributions of co-authors and co-researchers.
5) The results are appropriately placed in the context of prior and existing research.
6) All sources used are properly disclosed (correct citation). Literally copying of text must be indicated as such by using quotation marks and giving proper reference.
7) All authors have been personally and actively involved in substantial work leading to the paper, and will take public responsibility for its content.
8) Informed consent was obtained from cyclists who were surveyed. Consequently, in order to protect their information, we assure that the database available for this paper excludes any names and any type of personal information coming from them.

## Declaration of Competing Interest

None.

## Footnotes

1 Medellin is divided into 16 administrative neighborhoods. In this case, these strata correspond to the set of different neighborhoods located in the same sector. In this sense, stratum 1 corresponds to the 1^st^ to 4^th^ neighborhoods. The second stratum includes the 5^th^, 6^th^, and 7^th^ neighborhoods. The third stratum includes the 8^th^, 9^th^ and 10^th^ neighborhoods. The fourth stratum includes the 11^th^, 12 ^th^, and 13 ^th^ neighborhoods. The fifth stratum only includes the 14^th^ neighborhood, and finally, the sixth stratum includes the 15^th^ and 16^th^ neighborhoods.

2 The income level strata are a Colombian government tool used to classify real estate properties according to the categories of the national department of statistics (DANE). These strata evaluate real estate units based on poverty level, public services, location, indigenous population and other factors. This classification determines the level of taxation, fares for public services (water, energy, phone and gas), access to free health services, fares at public universities, poverty alleviation programs, etc. In most cases, income levels 1 and 2 receive subsidies from the upper levels 4, 5 and 6. Although this tool is based on solidarity principles, it has a direct impact on urban morphology and the location of activities and population in Colombian cities [7].
